# Cariprazine for Treating Schizophrenia, Mania, Bipolar Depression, and Unipolar Depression: A Review of Its Efficacy

**DOI:** 10.7759/cureus.39309

**Published:** 2023-05-21

**Authors:** Martin Tarzian, Mariana Ndrio, Srujan Kaja, Elisabeth Beason, Adegbenro O Fakoya

**Affiliations:** 1 Psychiatry, University of Medicine and Health Sciences, Basseterre, KNA; 2 Psychiatry and Behavioral Sciences, University of Medicine and Health Sciences, Basseterre, KNA; 3 Psychiatry, Larkin Community Hospital, Miami, USA; 4 Cardiology, University of Medicine and Health Sciences, Basseterre, KNA; 5 Cellular Biology and Anatomy, Louisiana State University Health Sciences Center, Shreveport, USA

**Keywords:** psychiatric disorders, bipolar depression, unipolar depression, major depression, schizophrenia, cariprazine

## Abstract

This drug review presents a comprehensive review of Cariprazine, a medication that received FDA approval in 2015 for treating schizophrenia and bipolar disorder. The paper begins by exploring Cariprazine's mechanism of action, which involves modulating dopamine and serotonin receptors. Additionally, the review assesses Cariprazine's metabolic profile and notes its low potential for weight gain and metabolic side effects. The study examines Cariprazine's efficacy and safety in treating various psychiatric disorders, such as schizophrenia, bipolar maintenance, mania, and bipolar depression. A meticulous analysis of clinical trials is included, demonstrating Cariprazine's potential advantages over existing medications used for these disorders. Additionally, the review covers Cariprazine's recent approval as an adjuvant treatment for unipolar depression. Furthermore, the paper examines the limitations of Cariprazine, such as the absence of head-to-head trials comparing it to other commonly used medications for these disorders. The paper concludes by emphasizing the need for more research to establish Cariprazine's position in treating schizophrenia and bipolar disorder and determine its comparative effectiveness with other available treatments.

## Introduction and background

Psychotic disorders such as schizophrenia and mood disorders such as major depression and bipolar disorder are complex and multifaceted mental illnesses that can severely impair an individual's daily functioning and quality of life [1,2]. According to the Institute of Health Metrics and Evaluation's latest data, in 2019, approximately 280 million individuals worldwide lived with depression, including 23 million children and adolescents, while 40 million people lived with bipolar depression [3-5]. Although schizophrenia is not as prevalent as other psychiatric disorders, affecting around 24 million individuals globally, the World Health Organization considers it one of the top 10 illnesses that significantly contribute to the global burden of disease, with devastating outcomes for affected individuals, including financial ruin [3-6].

Previously, managing major depression, bipolar disorders, and schizophrenia required different pharmacological treatments, with antidepressants, mood stabilizers, and antipsychotics used to address each disorder [7]. While first-generation antipsychotics were the gold standard for treating schizophrenia, a shift in treatment paradigm occurred in the late 1990s with the emergence of novel atypical antipsychotic agents [8]. These newer agents had a more favorable safety and tolerability profile than the first-generation antipsychotics, attributed to their less specific antagonistic activity at the D2 receptor and relatively stronger serotonin 5-HT2A receptor action [9]. This mechanism not only reduced the potential for developing extrapyramidal symptoms (EPS), which was a treatment-limiting side effect of first-generation antipsychotics, but also allowed these agents to be used in the treatment of a broader range of conditions other than in schizophrenia, including bipolar disorder and major depression, where they can be used as an adjunctive therapy or even a monotherapy [9]. A study revealed that atypical antipsychotics were prescribed to more than 70% of patients with nonpsychotic conditions such as anxiety disorders and bipolar disorder for mood stabilization [9].

According to data collected from the National Health and Nutrition Examination Survey spanning from 2013 to 2018, approximately 3.8 million adults in the United States, or 1.6% of the population, were found to be taking antipsychotic medications [10]. Despite their efficacy, significant concerns exist regarding these agents' side effect profiles, including weight gain, metabolic syndrome, and cardiovascular complications [8]. At the same time, while at a lesser degree, these agents still possess the potential to predispose to EPS. As a result, pharmaceutical research has shifted its focus toward identifying newer atypical antipsychotics that exhibit a more favorable profile regarding adverse effects.

Cariprazine (brand name Vraylar) is a more recent addition to a unique group of antipsychotics with a receptor profile characterized by dopamine D3-preferring D3/D2 receptor partial agonism and serotonin 5-HT1A partial agonism [11,12]. Studies have shown that cariprazine has a lower risk of causing significant weight gain, hyperlipidemia, and hyperglycemia [11,12]. Due to this mechanism, cariprazine received FDA approval in 2015 for treating schizophrenia and acute manic or mixed episodes associated with bipolar I disorder, and in 2022, it received FDA approval as an adjunctive therapy for treating major depressive disorder [13].

This pharmacological review provides an in-depth examination of cariprazine, focusing on its mechanism of action, metabolic profile, clinical effectiveness, and safety in treating various mental illnesses. The article evaluates the evidence of cariprazine's potential advantages over existing pharmaceuticals and its limitations, including the absence of head-to-head trials comparing it to other commonly used therapies. Finally, the study comprehensively overviews cariprazine's role in treating schizophrenia and bipolar disorder. It highlights areas for further research to determine comparative efficacy and appropriate therapeutic usage.

## Review

Method

For this drug review on Vraylar and its trials, Google Scholar was used to search for relevant research papers, clinical trials, and other scientific articles related to Vraylar. The search terms included "Vraylar," "cariprazine," "clinical trials," "bipolar mania," "bipolar depression," and "schizophrenia." The inclusion criteria for the articles were that they had to be published in peer-reviewed journals and had to report on the efficacy and safety of Vraylar in treating schizophrenia or bipolar disorder. The exclusion criteria were articles that were not related to the topic, duplicate publications, and articles that were not in English. After a thorough search of the literature, a total of 48 relevant articles were selected for the review. The articles were read and analyzed for content, and key findings were extracted. The extracted data included the study design, sample size, inclusion and exclusion criteria, interventions, outcome measures, and results. The ChatGPT language model (OpenAI, San Francisco, California) was utilized to assist in editing and rewording the work to ensure coherence and clarity in conveying the findings. In summary, a comprehensive review of relevant literature on Vraylar was conducted using Google Scholar, and Chat GPT was utilized to assist in editing. The review included an analysis of the study design, sample size, interventions, outcome measures, and results of 48 selected articles related to the efficacy and safety of Vraylar in treating schizophrenia and bipolar disorder.

Mechanism of action

Cariprazine is a unique antipsychotic drug that antagonizes D3 receptors while blocking D2 receptors. Cariprazine is the only antipsychotic medication targeting dopamine D3 receptors [13,14]. While other antipsychotic medications may have some affinity for the D3 receptor, they primarily target other dopamine receptors, such as D2 [14-16].

D3 receptor antagonists have been shown to have potential pro-cognitive effects, including improvements in attention, working memory, and executive function, in animal models and clinical studies [17,18]. The clinical significance of cariprazine's unique targeting of D3 receptors is not fully understood; however, preclinical studies suggest that D3 receptor blockade may have potential therapeutic benefits in treating schizophrenia and other psychiatric disorders [15].

Cariprazine also has a relatively high affinity for 5HT1A and alpha 1B receptors [11,14,15]. This pharmacological profile is thought to contribute to its potential efficacy and tolerability in treating schizophrenia and related disorders. Specifically, high affinity at the 5HT1A receptor is associated with improved negative symptoms and cognitive deficits [15].

The 5HT1A receptor is a subtype of serotonin receptor widely distributed throughout the brain and involved in various physiological and psychological processes, including mood, cognition, and stress responses [19,20]. Activation of the 5HT1A receptor has been shown to have potential antidepressant and anxiolytic effects and pro-cognitive effects in animal models and clinical studies [19,20]. Cariprazine's high affinity at the 5HT1A receptor may contribute to its potential efficacy in improving negative symptoms and cognitive deficits in patients with schizophrenia and bipolar depression [15].

The alpha 1B receptor is a subtype of alpha-adrenergic receptor that is primarily expressed in the central nervous system and is involved in regulating motor function and cardiovascular activity. Alpha 1B receptor antagonists have been shown to have antipsychotic effects and reduce EPS [16,20]. Cariprazine's high affinity at the alpha 1B receptor may contribute to its potential tolerability in treating schizophrenia and related disorders [11,12,15]. In contrast, high affinity at the alpha 1B receptor is hypothesized to be linked to reduced EPS, such as parkinsonism and akathisia [16,20].

Metabolic profile and tolerability

Unlike many other antipsychotics, studies have demonstrated that cariprazine stands out from other antipsychotic medications due to its favorable metabolic profile. It carries a lower risk of causing common metabolic side effects such as significant weight gain, hyperlipidemia, and hyperglycemia [15,21]. Clinical studies have reported that cariprazine is associated with minimal changes in body weight, BMI, and waist circumference, even during long-term treatment periods of up to 48 weeks [22-24]. Cariprazine was also shown to have a lower incidence of metabolic abnormalities such as hyperglycemia and hyperlipidemia when compared to risperidone [21,23].

The unique pharmacological properties of cariprazine, including its partial agonist activity at the dopamine D3 receptor and low affinity for the histamine H1 receptor, are thought to contribute to its favorable metabolic profile [15,21]. Additionally, cariprazine has been shown to have a low risk of causing metabolic abnormalities such as hyperlipidemia and hyperglycemia. In a study comparing cariprazine to risperidone, cariprazine was associated with significantly lower increases in fasting glucose and triglyceride levels and a lower incidence of hyperglycemia and hyperlipidemia [23].

Caccia et al. (2013) performed a thorough review of cariprazine and its side effect profile compared to other antipsychotics. Cariprazine showed good tolerability in studies involving schizophrenic patients [21]. The most commonly reported adverse events (AEs) with cariprazine included insomnia, extrapyramidal disorder, sedation, akathisia, nausea, dizziness, vomiting, anxiety, and constipation [21]. These side effects, however, occur at significantly lower rates than in other antipsychotics, such as olanzapine and risperidone [22,23]. In addition, there were no clinically significant changes observed in metabolic variables, prolactin levels, or corrected QT (QTc) prolongation (above 500 ms) with cariprazine use. Weight gain was found to be lower with cariprazine compared to risperidone and placebo [21]. Cariprazine shows minimal changes in blood pressure, pulse, and body weight observed in cariprazine-treated patients compared to placebo [21]. The incidence of orthostatic hypotension or ECG changes is similar between cariprazine and placebo groups. No cariprazine recipients had an ECG QT interval exceeding 500 ms [21].

Cariprazine may cause similar side effects as other second-generation antipsychotics (SGAs) but at a much lower rate [22,23]. Cariprazine, compared to first-generation antipsychotics and certain SGAs like olanzapine and risperidone, appears to have a lower prevalence of akathisia, a well-known side effect associated with antipsychotic treatment [23]. The distress caused by antipsychotic-induced akathisia can be significant and impact treatment adherence and long-term patient outcomes. However, in clinical practice, akathisia is routinely managed alongside other diseases or drug-induced symptoms.

In a pooled 48-week dataset, akathisia was the most commonly observed treatment-emergent adverse event (TEAE) with cariprazine. Most occurrences were reported as mild to moderate in severity and were considered related to treatment [22]. Akathisia rarely led to treatment discontinuation, with only a small percentage of patients discontinuing due to this side effect. The onset of akathisia generally occurs within the first six weeks of treatment [22]. In long-term cariprazine treatment, the majority of patients used medication to manage akathisia symptoms, regardless of the event's severity. This indicates that most patients were able to effectively manage akathisia while continuing treatment with cariprazine [22]. Overall, these findings suggest that cariprazine causes fewer cases of akathisia and that the majority of patients can effectively manage this side effect while continuing their treatment with cariprazine.

Based on the results of two long-term open-label studies, cariprazine demonstrated a generally safe and well-tolerated profile. Mean prolactin levels decreased across all dose groups. There were no significant changes in aminotransferase levels or alkaline phosphatase, and no dose-response relationship was observed [23]. Metabolic parameters showed insignificant changes, with mean total cholesterol, low-density lipoprotein, and high-density lipoprotein levels decreasing. There was no clear dose-response relationship observed for these metabolic parameters. The mean change in body weight was 1.58 kg, with 27% of patients experiencing weight gain and 11% experiencing weight loss of 7% or more [23].

Cardiovascular parameters, including blood pressure and pulse, did not show clinically significant changes. The most common EPS-related TEAEs, reported in at least 5% of patients, were akathisia, tremor, restlessness, and extrapyramidal disorder. In conclusion, these post hoc pooled analyses support the safety and tolerability of cariprazine within the FDA-recommended dose range of 1.5-6 mg/day [23].

In conclusion, cariprazine offers a promising option for patients with schizophrenia, as it is associated with minimal risk of causing weight gain and other metabolic side effects frequently seen with other antipsychotics. While the underlying mechanisms of cariprazine's metabolic effects require further investigation, its unique pharmacological profile provides insight into its favorable profile.

Cariprazine and treating schizophrenia-related psychosis

Cariprazine has emerged as a promising option for treating schizophrenia. The efficacy and safety of cariprazine in schizophrenia have been evaluated in numerous short-term and long-term clinical trials, which have shown significant improvements in symptomatology while maintaining good tolerability with minimal side effects. These clinical studies are discussed in this section, and Table 1 below summarizes the main clinical findings.

**Table 1:** Studies investigating the treatment of schizophrenia with cariprazine PANSS: positive and negative syndrome scale, CGI-S: clinical global impression-severity scale, AEs: adverse events

Clinical trial	Number of participants	Length of study	Type of study	Endpoint measurements	Treatment arms	Results	Side effects
Durgam et al, 2016 [25]	375	Six weeks	Randomized, double-blind, placebo-controlled, proof-of-concept trial	PANNS CGI-S	Low-dose cariprazine (1.5–4.5 mg/d), high-dose (6–12 mg/d), placebo	No significant differences between the two doses of cariprazine and placebo in PANSS total score, change, low-dose cariprazine showed significantly greater reductions in PANSS scores compared to placebo	Cariprazine was well-tolerated -Similar percentages of AEs in all groups (moderate and mild in severity), most common AEs akathisia (in both groups), restlessness tremor, back pain, and extrapyramidal disorder
Durgam et al, 2014 [26]	732	Six weeks	Randomized, double-blind, placebo- and active-controlled, fixed-dose trial	PANNS CGI-S	Cariprazine at 1.5 mg/d, 3.0 mg/d, or 4.5 mg/d, risperidone at 4.0 mg/d (for assay sensitivity), placebo	Reduction in PANSS and CGI-S scores compared to placebo for all active treatments, with the greatest improvement observed with cariprazine 4.5 mg/d	Common AEs were insomnia, extrapyramidal disorder, akathisia, sedation, nausea, dizziness, and constipation. Mean changes in metabolic parameters were small and similar between treatment groups
Durgam et al, 2015 [27]	617	Six weeks	Randomized, double-blind, placebo- and active-controlled, fixed-dose trial	PANNS CGI-S	Placebo, cariprazine 3 mg/d, cariprazine 6 mg/d, aripiprazole 10 mg/d	Cariprazine 3 and 6 mg/d showed improvements relative to placebo in PANSS total and CGI-S scores	Common AEs were Insomnia (all groups), akathisia (cariprazine 6 mg/d), and headache (placebo, cariprazine 6 mg/d)
Kane et al., 2015 [24]	446	Six weeks	Randomized, double-blind, placebo- and active-controlled, fixed-dose trial	PANNS CGI-S	Cariprazine 3-6 mg/d, cariprazine 6-9 mg/d, placebo	Improvement in PANSS and CGI-S total scores in the cariprazine arms compared to placebo	Cariprazine was well-tolerated, common AEs in both cariprazine groups were akathisia, extrapyramidal disorder, and tremor; most were mild to moderate in severity. Mean metabolic changes were small and similar between groups. Prolactin levels decreased in all groups
Durgam, 2016 [28]	765	97 weeks	Double-blind, placebo-controlled, parallel-group study	Time to relapse (worsening of symptom scores, psychiatric hospitalization, aggressive/violent behavior, or suicidal risk)	Flexible or fixed doses of cariprazine (3, 6, or 9 mg/d, depending on the open-label phase-fixed-dose cariprazine or placebo during the double-blind phase	Time to relapse was significantly longer in patients treated with cariprazine than in those treated with a placebo, long-term treatment with cariprazine was safe and could be used to prevent relapse in schizophrenia	AEs during open-label treatment: akathisia insomnia headache, no cariprazine-related AEs observed during double-blind treatment
Durgam, 2017 [29]	93	48 weeks	Open-label, flexible-dose trial	PANSS CGI-S	Cariprazine (1.5-4.5 mg/day)	Long-term cariprazine treatment was deemed safe and well-tolerated with no new safety concerns arising from long-term treatment	The most common AEs were akathisia, insomnia, and weight gain, with serious AEs occurring in 13%. Changes in metabolic parameters were minimal and not clinically significant. No prolactin elevation or significant changes in cardiovascular parameters
Cutler, 2018 [30]	586	53 weeks	Open-label, flexible-dose trial	PANSS CGI-S	Cariprazine doses ranging from 3 to 9 mg/d	Long-term treatment with cariprazine at doses up to 9 mg/d was safe and well tolerated in patients with schizophrenia, measures of efficacy remained stable	No unexpected safety issues or deaths were reported. The most commonly observed AEs were akathisia, headache, insomnia, and weight gain. Mean changes in metabolic, hepatic, and cardiovascular parameters were not clinically relevant. Mean body weight increased by 1.5 kg. Prolactin levels decreased slightly

In 2006, Durgam et al. at the Forest Research Institute conducted a phase 2 (NCT00404573) proof-of-concept trial to evaluate the safety and efficacy of cariprazine in treating acute exacerbation of schizophrenia [25]. The six-week double-blind, placebo-controlled trial involved 375 participants who were randomly assigned to receive either low-dose cariprazine (1.5-4.5 mg/day), high-dose (6-12 mg/day), or placebo. The primary efficacy measure was the change in the Positive and Negative Syndrome Scale (PANSS) total score, with the clinical global impression-severity (CGI-S) scale used as a secondary measure. While there were no significant differences between the two doses of cariprazine in PANSS total score, low-dose cariprazine showed significantly more significant reductions in PANSS total and PANSS negative scores compared to the placebo. High-dose cariprazine had similar findings when compared to placebo. Notably, the study showed similar percentages of patients in all three treatment groups reporting TEAEs. Most TEAEs were mild to moderate in intensity. The only TEAEs reported in at least 5% of patients in either cariprazine group and with an incidence of at least twice the rate in the placebo group during the double-blind treatment were akathisia (in both groups), restlessness (in the 1.5-4.5 mg/day group), tremor (in the 1.5-4.5 mg/day group), back pain (in the 1.5-4.5 mg/day group), and extrapyramidal disorder (in the 6-12 mg/day group) [25].

The findings suggest that treatment with cariprazine does not result in significantly different rates of TEAEs compared to placebo, with most TEAEs being mild to moderate in intensity. However, specific side effects such as akathisia, restlessness, tremor, and back pain in the cariprazine groups highlight the need for further investigation into the EPS side effects of this medication. Notably, the incidence of extrapyramidal disorder was higher in the higher dose group (6-12 mg/day) compared to the lower dose groups (placebo and 1.5-4.5 mg/day). This information is relevant for clinicians when considering the optimal dosing strategy for their patients. Additionally, further research could explore the potential risk factors or predictors of developing EPS side effects with cariprazine treatment. Such investigation could provide valuable information for clinicians and patients in making informed decisions about the benefits and risks of this medication.

In 2017, Earley et al. conducted a groundbreaking multinational study (NCT00694707) to evaluate the safety and efficacy of cariprazine compared to placebo and risperidone in treating acute exacerbation of schizophrenia. This double-blind, randomized, placebo- and active-controlled, fixed-dose trial enrolled 732 patients who received six-week treatment with a two-week safety follow-up period [26]. The participants were randomly assigned to receive either placebo, cariprazine at 1.5 mg/d, 3.0 mg/d, or 4.5 mg/d, or risperidone at 4.0 mg/d. The study's primary outcome measure was the improvement in PANSS total score at week six, which was statistically significant compared to placebo for all active treatments. The most remarkable improvement was observed with cariprazine 4.5 mg/d, which substantially reduced the PANSS total score of -10.4 [26]. Significant improvement was also observed in CGI-S for all active treatments. The most common AEs reported were insomnia, extrapyramidal disorder, akathisia, sedation, nausea, dizziness, and constipation. However, mean changes in metabolic parameters were generally small and similar between treatment groups, suggesting that cariprazine may have a favorable metabolic profile compared to other antipsychotics [26]. Overall, this landmark study provides strong evidence for the efficacy and safety of cariprazine in treating acute exacerbation of schizophrenia, with the highest dose (4.5 mg/d) showing the most significant improvement in symptoms.

Between April 2010 and December 2011, Durgam et al. conducted a double-blind, randomized, placebo- and active-controlled, fixed-dose study (NCT01104766) in multiple countries. Participants were randomly assigned to receive either a placebo (n = 153) cariprazine 3 mg/d (n = 155), cariprazine 6 mg/d (n = 157), or aripiprazole 10 mg/d (n = 152) for six weeks of treatment. The primary endpoint was the mean change from baseline to week six in PANSS total score, with the secondary endpoint being the CGI-S score. Results showed that both cariprazine 3 and 6 mg/d demonstrated statistically significant improvements relative to placebo in PANSS total score change at week six (3 mg/d, -6.0; 6 mg/d, -8.8) and CGI-S scores (3 mg/d, -0.4; 6 mg/d, -0.5). Similarly, aripiprazole showed significant differences from the placebo on the PANSS (-7.0) and CGI-S (-0.4). The most common TEAEs (≥ 10%) were insomnia (all groups), akathisia (cariprazine 6 mg/d), and headache (placebo, cariprazine 6 mg/d). Overall, this study supports the efficacy, safety, and tolerability of cariprazine 3 and 6 mg/d for treating acute exacerbation of schizophrenia [27].

In 2010, Kane et al. conducted a six-week, double-blind, placebo-controlled flexible-dose study (NCT01104779) with 446 participants randomized to receive a placebo, cariprazine 3 to 6 mg/d, or cariprazine 6 to 9 mg/d treatments [24]. The study evaluated primary and secondary efficacy measures, including changes from baseline to week six in PANSS total and CGI-S. The results demonstrated significant improvements compared to placebo for cariprazine 3 to 6 and 6 to 9 mg/d on the PANSS total score (3-6 mg/d: −6.8, 6-9 mg/d: −9.9) and CGI-S (3-6 mg/d: −0.3, P=0.012; 6-9 mg/d: −0.5) at week 6 [24]. Cariprazine was well-tolerated, and common TEAEs in both cariprazine groups were mostly mild to moderate in severity and included akathisia, extrapyramidal disorder, and tremor. Mean changes in metabolic parameters were generally small and similar between groups, and prolactin levels decreased in all groups [24]. This study further supports the potential of cariprazine 3 to 6 and 6 to 9 mg/d as an efficacious treatment for the acute exacerbation of schizophrenia [24].

In a long-term study conducted by Durgam et al. (NCT01412060), the efficacy, safety, and tolerability of cariprazine were evaluated for preventing relapse in adults with schizophrenia [28]. This multi-country, double-blind, placebo-controlled study was conducted for up to 97 weeks. It consisted of five distinct phases: screening, open-label run-in, stabilization, double-blind treatment (up to 72 weeks), and safety follow-up. Patients were administered flexible or fixed doses of cariprazine (3, 6, or 9 mg/d, depending on the open-label phase) and subsequently received either fixed-dose cariprazine or placebo during the double-blind phase. Of the 765 patients, 264 completed open-label treatment, and 200 eligible patients were randomized to a double-blind placebo (n=99) or cariprazine (n=101). The study showed that patients treated with cariprazine had a significantly longer time to relapse than those treated with placebo, with relapse occurring in only 24.8% of cariprazine-treated patients versus 47.5% of placebo-treated patients. During the open-label treatment, the most commonly reported AEs were akathisia (19.2%), insomnia (14.4%), and headache (12.0%). However, no cariprazine-related AEs of at least 10% were observed during double-blind treatment. The study concluded that long-term treatment with cariprazine was more effective than placebo in preventing relapse in patients with schizophrenia [28].

After successfully demonstrating the safety and efficacy of cariprazine in short-term clinical trials involving patients with acute exacerbation of schizophrenia, Durgam et al. conducted an open-label extension study (NCT00839852) to assess the long-term safety and tolerability of cariprazine in patients with schizophrenia [29]. This study enrolled patients who had completed the previous six-week, randomized, placebo- and active-controlled trial and had responded positively to treatment. Ninety-three patients received flexibly dosed, open-label cariprazine (1.5-4.5 mg/day) for up to 48 weeks, with almost half of them completing the full duration of treatment [29]. The study's most commonly reported AEs were akathisia, insomnia, and weight gain, with 13% of patients experiencing serious AEs and 11% discontinuing treatment due to AEs. Changes in metabolic parameters were minimal and not clinically significant, and no patient discontinued treatment due to changes in metabolic parameters or body weight [29]. Using cariprazine did not lead to prolactin elevation or significant changes in cardiovascular parameters. Overall, long-term treatment with cariprazine was considered safe and well-tolerated in this 48-week, single-arm trial, with no new safety concerns emerging from long-term treatment [29].

Cutler et al. conducted a multicenter, open-label, flexible-dose study to evaluate the safety and tolerability of cariprazine in patients with schizophrenia for 53 weeks (NCT01104792) [30]. The trial included both new patients and those who had participated in one of two phase 3 lead-in studies (NCT01104766, NCT01104779), and 586 patients were treated with cariprazine. The completion rate was approximately 39%, and there were no unexpected safety issues or deaths. The most common AEs were akathisia (16%), headache (13%), insomnia (13%), and weight gain (10%). Serious AEs occurred in 59 (10.1%) patients, and 73 (12.5%) patients discontinued the study due to AEs during open-label treatment [30]. The changes in metabolic, hepatic, and cardiovascular parameters were not clinically significant. The mean body weight increased modestly by 1.5 kg during the study, while prolactin levels decreased slightly, and efficacy measures remained stable [30]. The study concluded that cariprazine treatment up to 9 mg/d was generally safe and well tolerated in patients with schizophrenia over a long-term period [30].

It is important to note that researchers at the Forest Research Institute, an Allergan affiliate and manufacturer of cariprazine, conducted these clinical trials. However, it is worth mentioning that these studies were conducted per Good Clinical Practice guidelines and underwent rigorous evaluation by regulatory agencies, such as the FDA, before approval. Overall, the available evidence suggests that cariprazine is a safe and effective treatment option for schizophrenia, but further research is needed to understand its long-term safety and efficacy fully. Table 1 summarizes the studies investigating cariprazine for treating Schizophrenia.

Cariprazine for treating acute mania

Cariprazine has demonstrated remarkable efficacy in treating mania associated with bipolar disorder, as evidenced by multiple clinical trials yielding consistently positive outcomes.

Durgam et al. (2015) conducted a phase 2 trial to evaluate the efficacy, safety, and tolerability of cariprazine in treating acute manic or mixed episodes associated with bipolar I disorder [31]. The study was multinational, randomized, double-blind, and placebo-controlled, involving 236 patients who received cariprazine at a flexible dose of 3-12 mg/day after a washout period. After three weeks of double-blind treatment, the primary and secondary efficacy parameters, the Young Mania Rating Scale (YMRS), and CGI-S scores were assessed. The study found that cariprazine significantly reduced YMRS and CGI-S scores compared to placebo, with each YMRS item showing a significant improvement from baseline to week 3 for cariprazine. Moreover, a higher percentage of cariprazine patients met YMRS response (48% versus 25%) and remission (42% versus 23%) criteria at week 3 than placebo patients [31].

The study found that patients treated with cariprazine had an overall mean daily dose of 8.8 mg/day and experienced common AEs such as extrapyramidal disorder, headache, akathisia, constipation, nausea, and dyspepsia. The discontinuation rate due to AEs was 14% for cariprazine patients compared to 10% for placebo patients. Changes in metabolic parameters were similar between groups, except for fasting glucose which increased significantly more in the cariprazine group than in the placebo group (p < 0.05). The Barnes Akathisia Rating Scale and Simpson-Angus Scale showed that cariprazine patients were more likely to experience treatment-emergent akathisia (22% versus 6% for placebo) and EPS (parkinsonism) (16% versus 1% for placebo) [31].

In the randomized, double-blind, placebo-controlled, multicenter, parallel-group phase 2/3 studies conducted by Earley et al. (2018), adult patients between 18 and 65 years old with bipolar I disorder were enrolled. Post hoc analyses evaluated the efficacy of cariprazine in treating manic episodes, as measured by YMRS outcomes, including response (defined as a ≥50% decrease in score), remission (defined as a total score of ≤12 and ≤8), cumulative remission, and global improvement. Additionally, the study investigated composite remission, defined as a YMRS total score of ≤12 plus a Montgomery-Asberg Depression Rating Scale (MADRS) total score of ≤12, as well as worsening/switch to depression, defined as a MADRS total score of ≥15, every week [32]

In the study by Earley et al. (2018), significant improvements were observed in every measure evaluated for cariprazine compared to placebo (P < .01 for all analyses), with a number needed to treat of ≤10 for each measure. Furthermore, there was no evidence of worsening or a switch to depression. Although the study had limitations, including post hoc analyses, short treatment duration, and the absence of an active comparator, the results suggest that cariprazine can provide clinically significant relief for manic symptoms in patients with bipolar I disorder. Additionally, it did not induce depressive symptoms [32].

McIntyre et al. (2019) conducted a study that aimed to compare the efficacy of cariprazine versus placebo in patients with bipolar I disorder and mixed features. The review included data from three studies that employed different criteria to determine efficacy, such as DSM-5 criteria and MADRS scores. The results indicated that cariprazine was significantly more effective than placebo in reducing YMRS scores, particularly in patients meeting the ≥10 MADRS criterion. Furthermore, cariprazine improved depressive symptoms in the ≥10 MADRS group. More patients taking cariprazine met response and remission criteria for YMRS, with the most significant results observed in the ≥10 MADRS group. However, the study had some limitations, including being a post hoc analysis and the entry criterion o the MADRS scores <18, which could potentially limit the assessment of MADRS changes. Overall, cariprazine showed efficacy in reducing both manic and depressive symptoms in patients with mixed features, with varying efficacy based on specific criteria [33].

In 2020, Pinto et al. conducted a rigorous meta-analysis to examine the efficacy of cariprazine, marketed as cariprazine, in treating bipolar disorder. The study meticulously analyzed data from seven randomized controlled trials, providing a comprehensive overview of the drug's therapeutic potential. The results of the analysis were striking; cariprazine was found to be associated with a moderate yet significant reduction in manic symptoms, as measured by the YMRS change scores. Moreover, the drug significantly increased remission and response rates for both manic and mixed episodes compared to the placebo. This finding underscores the potential of cariprazine as a promising treatment option for bipolar disorder, offering hope to individuals suffering from this debilitating condition [34].

The comprehensive meta-analysis conducted by Pinto et al. (2020) demonstrated the safety and effectiveness of cariprazine in treating acute manic and mixed episodes associated with bipolar disorder. In terms of depressive symptoms, the use of cariprazine at daily doses of 1.5 mg and 3 mg demonstrated a modest yet significant improvement, based on MADRS scores. Although the drug was associated with adverse effects, it did not result in higher dropout rates due to adverse effects compared to the placebo. In addition, the study revealed that cariprazine at doses of 1.5-3 mg daily is efficacious in treating acute bipolar depression, albeit with smaller effect sizes. The findings of this meta-analysis provide strong evidence for the use of cariprazine in managing the symptoms of bipolar disorder [34].

In 2015, Calabrese et al. conducted a phase 3 trial to assess the efficacy, safety, and tolerability of cariprazine in treating patients with acute manic or mixed episodes related to bipolar I disorder. The trial comprised 497 randomized into three groups: placebo, cariprazine 3-6 mg/d, or cariprazine 6-12 mg/d for three weeks of double-blind treatment [35].

The study found that low- and high-dose cariprazine was significantly more effective than placebo in reducing manic symptoms, as assessed by the YMRS total score. The difference in the least squares means change from baseline to week 3 in the YMRS total score favored both cariprazine treatment groups over placebo (LSMD [95% CI]: 3-6 mg/d, -6.1 [-8.4 to -3.8]; 6-12 mg/d, -5.9 [-8.2, -3.6]; P < .001 [both]). Additionally, both cariprazine treatment groups showed statistically significant superiority to the placebo on all 11 YMRS single items [35].

In addition to reducing manic symptoms, the study found that cariprazine treatment was associated with significant improvement in the CGI-S of Illness scores when compared to placebo. Treatment-related AEs, including akathisia, nausea, constipation, and tremor, were generally manageable, with akathisia being the most common. Despite this, cariprazine was both effective and well-tolerated for treating acute manic or mixed episodes associated with bipolar I disorder, with the caveat that the incidence of akathisia was higher in the cariprazine group than in the placebo group [35].

Overall, the collective evidence from multiple randomized controlled trials supports the effectiveness of cariprazine in treating mania associated with bipolar disorder. These studies have consistently demonstrated significant improvements in manic symptoms, with fewer AEs. Additionally, cariprazine has shown promise in preventing the recurrence of manic or mixed episodes over the long term. These findings underscore the potential value of cariprazine as a treatment option for patients with bipolar disorder who experience manic or mixed episodes. Table 2 summarizes the studies investigating the treatment of mania with cariprazine.

**Table 2:** Studies investigating the treatment of mania with cariprazine YMRS: Young Mania Rating Scale, CGI-S: clinical global impression-severity scale, MADRS: Montgomery-Asberg depression rating scale, EPS: extrapyramidal symptoms, AEs: adverse events

Clinical trial	Number of participants	Length of study	Type of study	Endpoint measurements	Treatment arms	Results	Side effects
Durgam et al., 2015 [31]	236	Three weeks	Double-blind, placebo-controlled study	YMRS, CGI-S	Cariprazine at a flexible dose of 3-12 mg/day after washout, placebo	Cariprazine was found to significantly reduce manic or mixed episodes associated with bipolar I disorder, as measured by YMRS and CGI-S scores. A higher percentage of patients treated with cariprazine met YMRS response and remission criteria at week 3 compared to placebo patients.	Common AEs associated with cariprazine included extrapyramidal disorder, headache, akathisia, constipation, nausea, and dyspepsia, and treatment-emergent akathisia and EPS (parkinsonism) were more likely to occur in cariprazine patients.
Earley et al., 2017 [32]	1065	Three weeks	Post hoc analysis of three randomized, double-blind, placebo-controlled, multicenter, parallel-group phase 2/3 studies	YMRS	In two of the trials (RGH-MD-31 and RGH-MD-32), a flexible-dose design with cariprazine doses of 3-12 mg/d was used; in the third trial (RGH-MD-33), a fixed/flexible-dose design with two cariprazine-treatment arms (3-6 mg/d or 6-12 mg/d) was used. All cariprazine doses were pooled for post hoc analyses.	Cariprazine was found to be significantly more effective than placebo in every measure evaluated (P < .01 for all analyses), with a number needed to treat of ≤10 for each measure. Cariprazine did not induce depressive symptoms and may provide clinically significant relief for manic symptoms in patients with bipolar I disorder.	None
McIntyre et al., 2019 [33]	1037	Three weeks	Post hoc analysis of data from three similarly-designed trials of cariprazine compared to placebo	MADRS, YMRS	Placebo, cariprazine 3.0-6.0 mg/d, or 6.0-12.0 mg/d	Cariprazine was significantly more effective than placebo in reducing YMRS scores, particularly in patients meeting the ≥10 MADRS criterion. Cariprazine improved depressive symptoms in the ≥10 MADRS group. More patients taking cariprazine met response and remission criteria for YMRS, with the most significant results observed in the ≥10 MADRS group.	None
Pinto et al., 2020 [34]	Seven studies	Numerous timelines were used in the meta-analysis	Meta-analysis	MADRS, YMRS	Numerous doses of cariprazine were compared to a placebo in this meta-analysis	The study showed that cariprazine is safe and effective in treating acute manic and mixed episodes associated with bipolar disorder. The study also found that cariprazine at 1.5-3 mg daily can effectively treat acute bipolar depression, although with smaller effect sizes. The meta-analysis provides strong evidence for the use of cariprazine in managing the symptoms of bipolar disorder.	None
Calabrese et al., 2014 [35]	497	Three weeks	Double-blind, placebo-controlled study	YMRS, CGI-S	Placebo, cariprazine 3-6 mg/d, or cariprazine 6-12 mg/d	Calabrese et al. conducted a phase 3 trial in 2014 to assess the efficacy, safety, and tolerability of cariprazine in treating acute manic or mixed episodes related to bipolar I disorder. The study found that low- and high-dose cariprazine was significantly more effective than placebo in reducing manic symptoms, as measured by the YMRS total score, and also demonstrated significant improvement in the CGI-S scores compared to placebo. Despite the manageable incidence of treatment-related AEs, such as akathisia, nausea, constipation, and tremor, cariprazine was effective and well-tolerated for treating acute manic or mixed episodes associated with bipolar I disorder.	The most common treatment-related AEs associated with cariprazine were manageable and included akathisia, nausea, constipation, and tremor.

Cariprazine for treating bipolar depression

Bipolar depression is a common and debilitating condition affecting millions worldwide. Cariprazine has been studied extensively for its efficacy in treating bipolar depression. In this section, we will review the clinical trials that demonstrated the efficacy of cariprazine in treating bipolar depression.

In 2009, Yatham et al. conducted a phase 2 clinical trial (NCT00852202) to evaluate the efficacy, safety, and tolerability of cariprazine versus placebo for depressive episodes associated with bipolar I or II disorder (Yatham, 2020). This eight-week, double-blind, placebo-controlled, fixed/flexible-dose study enrolled 234 patients randomized in a 1:1:1 ratio to placebo, "low-dose" cariprazine (0.25-0.5 mg/day), or "high-dose" cariprazine (1.5-3.0 mg/day) [36]. The primary endpoint was the change in the MADRS total scores from baseline to week eight. The secondary endpoint was the mean clinical global impressions-improvement score at week eight. The study found that neither the low-dose nor high-dose cariprazine group showed significant differences from the placebo group. Nonetheless, this trial provided useful information that was utilized in the design and implementation of subsequent phase 2b/3 clinical trials of cariprazine in bipolar depression. No new safety concerns were observed with cariprazine, and the most common TEAEs (≥5% of cariprazine patients and twice the rate of placebo) included insomnia, akathisia, dry mouth, nausea, weight increase, diarrhea, restlessness, vomiting, musculoskeletal stiffness, migraine, and cough. Metabolic and weight changes were generally comparable between the cariprazine and placebo groups [36].

In 2016, Durgam et al. conducted a phase 2 clinical trial (NCT01396447) to evaluate the efficacy, safety, and tolerability of cariprazine for major depressive episodes associated with bipolar I disorder. The trial was a multinational, multicenter, double-blind, placebo-controlled, parallel-group, fixed-dose study, involving adult patients who received either placebo or cariprazine at doses of 0.75, 1.5, or 3.0 mg/day for eight weeks [37]. The study included 571 patients, and the primary and secondary efficacy endpoints were the change in the MADRS and CGI-S scores, respectively. Results showed that cariprazine at 1.5 mg/day significantly improved MADRS total score compared with placebo (least squares mean difference = -4.0, 95% CI=-6.3, -1.6; adjusted for multiple comparisons). However, cariprazine at 3.0 mg/day showed a reduction in the MADRS score that was not statistically significant when adjusted for multiple comparisons. The 0.75 mg/day dosage was similar to placebo. A similar pattern for significance was observed on the CGI-S. The most common AEs (≥10%) in cariprazine-treated patients were akathisia and insomnia, and weight gain was slightly higher with cariprazine than with placebo. The study concluded that cariprazine at 1.5 mg/day demonstrated consistent efficacy compared with placebo across outcomes and was generally well tolerated, suggesting its efficacy for treating bipolar I depression [37].

In 2016, a multinational, a six-week long, phase 3 double‐blind placebo‐controlled trial (NCT02670538) was conducted to evaluate the efficacy and safety of cariprazine in adult patients with bipolar I disorder and a current depressive episode [38]. A total of 493 patients were randomized to receive either placebo (n=167), cariprazine 1.5 mg/day (n=168), or cariprazine 3.0 mg/day (n=158). The primary efficacy endpoint was the change from baseline to week six in the MADRS total scores, and the secondary endpoint was the change in the CGI-S scores compared to the placebo (Earley, 2020). The results showed that cariprazine 1.5 mg/day significantly reduced depressive symptoms compared to placebo, both in primary (MADRS LSMD=-2.5; adjusted P=.0417) and secondary (CGI-S LSMD=-0.3; adjusted P=.0417) efficacy measures. However, the differences were not statistically significant for cariprazine 3.0 mg/day. The most common TEAEs were akathisia, restlessness, nausea, and fatigue. The study also found that the mean metabolic parameter changes were low and generally comparable among groups, and mean weight increases were ≤0.5 kg for all groups. Overall, the safety and tolerability profiles of cariprazine were similar to previous studies [38].

A subsequent phase 2 clinical trial was designed to build on the findings of a previous study and evaluate the efficacy and safety of cariprazine at doses of 1.5 mg/day and 3.0 mg/day versus placebo in treating adults with bipolar I depression (NCT02670551) [39]. The trial was a randomized, double-blind, fixed-dose study that included 488 patients randomized to receive either cariprazine 1.5 mg/day, cariprazine 3.0 mg/day, or a placebo. The primary efficacy endpoint was the change from baseline to week six in the MADRS total score. Both cariprazine groups showed significant reductions in MADRS total scores compared to placebo, with the 1.5 mg/day group showing a reduction of -2.5 (95% CI: -4.6 to -0.4; adjusted p = 0.033) and the 3.0 mg/day group showing a reduction of -3.0 (95% CI: -5.1 to -0.9; p = 0.010). MADRS response rates only reached statistical significance for the cariprazine 3.0 mg/day group (51.8%, NNT = 8, p = 0.024) (Earley, 2019). However, both cariprazine groups had significantly higher MADRS remission rates (33.1%, NNT = 10, p = 0.037 for the 1.5 mg/day group and 32.3%, NNT = 11, p = 0.039 for the 3.0 mg/day group) [39]. Overall, these findings were consistent with those of previous phase 2 and 3 trials, demonstrating the efficacy of both 1.5 mg/day and 3.0 mg/day doses of cariprazine in achieving the primary endpoint and lower CGI-S scores than placebo. Moreover, both doses showed good tolerability profiles, low discontinuation rates, and no clinically significant metabolic changes or weight gain [39].

A pooled post hoc analysis by Mcintyre et al. aimed to evaluate the efficacy of cariprazine in the treatment of bipolar depression with or without concurrent manic symptoms [40]. This study aimed to evaluate the efficacy of cariprazine in treating bipolar I depression with or without manic symptoms. The study included patients from three randomized, double-blind, placebo-controlled trials who met DSM-IV-TR or DSM-5 criteria for bipolar I disorder with a current major depressive episode and had concurrent manic symptoms by a baseline YMRS total score of least 4. The efficacy of cariprazine 1.5 and 3 mg/day doses was compared to placebo. The analysis included the least squares mean change from baseline to week six in the MADRS total score. The study included 1383 randomized to treatment, with 808 (58.4%) having concurrent manic symptoms. For patients with manic symptoms, cariprazine 1.5 and 3 mg/day significantly reduced the mean MADRS total score from baseline to week six compared to the placebo, respectively. For patients without manic symptoms, the LSMD was significant for 1.5 mg/day (-3.3; p = .0008) but not for 3 mg/day (-1.9; p = .0562). This post hoc analysis suggests that cariprazine may be an appropriate treatment option for patients with bipolar I depression with or without manic symptoms. Higher doses are potentially more effective in patients with manic symptoms [40].

Taken together, these five trials demonstrate the efficacy of cariprazine in treating bipolar depression. Cariprazine appears well-tolerated, with the most common AEs being akathisia, restlessness, and nausea. Further studies are needed to confirm these findings and to explore the long-term safety and efficacy of cariprazine in treating bipolar depression. Table 3 summarizes the studies investigating the treatment of bipolar depression with cariprazine. 

**Table 3:** Studies investigating the treatment of bipolar depression with cariprazine CGI-S: clinical global impression-severity scale, MADRS: Montgomery-Asberg depression rating scale, TEAEs: treatment-emergent adverse events, AEs: adverse events

Clinical trial identification number and reference	Number of participants	Length of study	Type of study	Endpoint measurements	Treatment arms	Results	Side effects
Yatham et al., 2020 [36]	234	Eight weeks	Double-blind, placebo study	MADRS CGI-S	Placebo, "low-dose" cariprazine (0.25-0.5 mg/day), or "high-dose" cariprazine (1.5-3.0 mg/day)	Neither the low-dose nor high-dose cariprazine group showed significant differences from the placebo group in the primary endpoint of change in MADRS total scores from baseline to week eight. The study helped to identify factors that may have influenced this outcome, which helped to design and conduct subsequent clinical trials of cariprazine in bipolar depression.	No new safety signals were observed with cariprazine, and the most common TEAEs were generally similar between the cariprazine and placebo groups. The most common TEAEs (≥5% of cariprazine patients and twice the rate of placebo) included insomnia, akathisia, dry mouth, nausea, weight increase, diarrhea, restlessness, vomiting, musculoskeletal stiffness, migraine, and cough.
Durgam et al., 2011 [37]	571	Eight Weeks	Double-blind, placebo-controlled, parallel-group, fixed-dose study	MADRS CGI-S	placebo or cariprazine at 0.75, 1.5, or 3.0 mg/day	Cariprazine at 1.5 mg/day significantly improved MADRS total score compared with placebo. Cariprazine at 3.0 mg/day showed a greater reduction in MADRS score than placebo, but it was not statistically significant when adjusted for multiple comparisons. The 0.75 mg/day dosage was similar to the placebo. A similar pattern for significance was observed on the CGI-S. The study concluded that cariprazine at 1.5 mg/day demonstrated consistent efficacy compared with placebo across outcomes and was generally well-tolerated, suggesting its efficacy for treating bipolar I depression.	The most common AEs (≥10%) in cariprazine-treated patients were akathisia and insomnia, and weight gain was slightly higher with cariprazine than with placebo.
Earley et al., 2020 [38]	493	Six weeks	Fixed-dose, randomized, controlled trial	MADRS CGI-S	cariprazine at 1.5 mg/day and 3.0 mg/day compared with placebo	-Cariprazine 1.5 mg/day significantly reduced depressive symptoms compared to placebo, both in primary (MADRS LSMD=-2.5; adjusted P=.0417) and secondary (CGI-S LSMD=-0.3; adjusted P=.0417) efficacy measures. However, the differences were not statistically significant for cariprazine 3.0 mg/day. -The study also found that the mean metabolic parameter changes were low and generally comparable among groups, and mean weight increases were ≤0.5 kg for all groups. -Overall, the safety and tolerability profiles of cariprazine were similar to previous studies.	The most common TEAEs were akathisia, restlessness, nausea, and fatigue.
Earley et al., 2019 [39]	488	Six weeks	Fixed-dose, randomized, controlled trial	MADRS CGI-S	cariprazine at 1.5 mg/day, 3.0 mg/day and placebo	-Both cariprazine doses significantly reduced MADRS total scores from baseline to week 6 compared to placebo -MADRS remission rates were significantly higher in both cariprazine groups, while response rates only reached statistical significance for the cariprazine 3.0 mg/day group -The study concluded that both 1.5 mg/day and 3.0 mg/day doses of cariprazine were effective in achieving the primary endpoint when compared to placebo, and CGI-S scores were lower in cariprazine groups compared to placebo	Cariprazine had good tolerability profiles, low rates of discontinuation, and no clinically significant metabolic changes or weight gain.
McIntyre et al., 2020 [40]	1383	Six weeks	Three randomized, double-blind, placebo-controlled trials	MADRS	cariprazine 1.5 and 3 mg/day doses were compared to placebo	-Cariprazine effectively reduced the MADRS total score in patients with bipolar I depression and concurrent manic symptoms compared to placebo. -Both 1.5 and 3 mg/day doses of cariprazine effectively reduced MADRS total score in patients with concurrent manic symptoms. -Cariprazine 1.5 mg/day also effectively reduced MADRS total score in patients without concurrent manic symptoms. -These results suggest that cariprazine may be a suitable treatment option for patients with bipolar I depression, with higher doses potentially being more effective in patients with concurrent manic symptoms.	None

At the time of writing, there are three clinical trials on the efficacy and safety of cariprazine in bipolar disorder that are currently ongoing and registered on ClinicalTrials.gov. One of these is a randomized controlled trial that aims to evaluate the efficacy and safety of cariprazine in treating pediatric patients with bipolar I depression (NCT04777357). Another is an open-label study that will assess the long-term safety and tolerability of cariprazine in pediatric patients with schizophrenia or bipolar I disorder for 26 weeks (NCT04578756). Lastly, a randomized controlled trial aims to investigate relapse prevention in bipolar I patients with manic or depressive episodes, with or without mixed features (NCT03573297). However, it is essential to note that the preliminary findings of these ongoing clinical trials are not yet available, and further research is needed to fully understand the safety and efficacy of cariprazine in the treatment of bipolar disorder.

Cariprazine as an adjuvant for treating unipolar depression

After a few phase 2 and phase 3 human clinical trials, cariprazine was recently approved for adjunctive treatment of major depressive disorder (MDD) in adults who had not responded adequately to antidepressant monotherapy [13]. Cariprazine had demonstrated antidepressant-like properties in several animal behavior models during preclinical trials [41]. This section will discuss some clinical trials evaluating cariprazine's efficacy in the treatment of unipolar depression.

In a randomized, double-blind, placebo-controlled, flexible-dose study, Durgam et al. investigated the efficacy and safety of adjunctive treatment with cariprazine in adults with MDD who had an inadequate response to standard antidepressants (NCT01469377) [42]. The study was conducted from December 2011 to December 2013. A total of 819 eligible patients were randomized to receive either placebo (n = 269), cariprazine 1-2 mg/day (n = 274), or cariprazine 2-4.5 mg/day (n = 276) for 8 weeks. The primary efficacy parameter was the change in the MADRS total score from baseline to week eight. The study found that adjunctive cariprazine 2-4.5 mg/day was significantly more effective than placebo in reducing MADRS total score at week eight (least squares mean difference [LSMD] = -2.2; adjusted P = .0114), but cariprazine 1-2 mg/day did not show significant improvement compared to placebo (LSMD = -0.9; adjusted P = .2404) [42]. Significant LSMDs were observed in the 2-4.5 mg/day group at all earlier study visits (weeks two, four, and six) and in the 1-2 mg/day group at weeks two and four (all P values < .05). The study concluded that adjunctive cariprazine 2-4.5 mg/day was effective and generally well-tolerated in adults with MDD who had inadequate responses to standard antidepressants [42].

After this trial, a phase 3 randomized, double-blind, placebo-controlled trial that lasted 18 to 19 weeks (NCT01715805) was conducted to further evaluate the efficacy of cariprazine (1.5-4.5 mg/day) as an adjunctive treatment to antidepressant therapy (ADT) for MDD [43]. In this study, during an eight-week open-label period, ADT response was assessed, and inadequate responders were randomized (N = 530) to open-label ADT plus placebo (n = 261) or cariprazine (n = 269) for the eight-week double-blind phase. Results showed that cariprazine did not significantly improve MADRS scores compared to placebo ([LSMD]: -0.2, P = 0.7948 and SDS LSMD: -0.7, P = 0.2784). However, cariprazine did improve CGI-I scores (LSMD: -0.2; P = 0.0410) versus placebo. The results of this study contrasted with previously published results [43].

To further evaluate the safety of cariprazine plus adjunctive ADT in patients with MDD, a long-term, open-label study (NCT01838876) was also carried out [44]. In this study, some participants had previously participated in the negative eight-week study above (n=311) (NCT01715805), while others were newly enrolled and had no prior exposure to cariprazine (n=131). Of the patients who continued in the study, a higher percentage of continuing patients (66.2%) completed the study than new patients with no prior exposure to cariprazine (35.9%). AEs were reported in 79% of patients, with the most common being akathisia (15.9%) and headache (11.6%). The mean changes in cardiovascular and ophthalmologic parameters were generally not clinically significant. The study showed that cariprazine was generally safe and well tolerated as adjunctive therapy to treat MDD, with a remission rate of 53.3% by week 26. Serious AEs occurred in 2% of patients, and two deaths occurred (one traffic accident and one completed suicide, both considered unrelated to treatment) [44].

Fava et al. also conducted a long term double-blind, placebo-controlled, randomized phase 2 study which lasted 19 weeks to evaluate the efficacy, safety, and tolerability of adjunctive cariprazine at doses of 0.1-0.3 and 1.0-2.0 mg/day for treating treatment-resistant MDD in adults (NCT00854100) [45]. A total of 231 patients were randomized. The primary and secondary endpoints were the change in MADRS CGI-S total scores. Although none of the predefined parameters reached significance for either dose of cariprazine, higher doses were associated with numerically more significant mean changes in MADRS and CGI-S scores and MADRS response and remission rates, respectively, to placebo [45]. No significant differences were observed between cariprazine 0.1-0.3 mg/day and placebo. Although not statistically significant, the MADRS response rates observed in patients taking cariprazine 1.0-2.0 mg/day exceeded the 10% threshold typically clinically significant [45]. The mean difference in responders between cariprazine and placebo (12.5%) was higher than the difference observed in the first trial for cariprazine 2.0-4.5 mg/day (11.1%) discussed above [42].

A post hoc analysis of a phase 3 clinical trial (NCT03738215) assessing the efficacy of cariprazine as an adjunctive to ADT in improving depressive symptoms in various patient subgroups was published by Papakostas et al. in 2023 [46]. The trial involved 759 patients who were randomized to receive placebo + ADT, cariprazine 1.5 mg/d + ADT, or cariprazine 3 mg/d + ADT for six weeks of double-blind treatment. The subgroups in this trial were categorized based on their level of response to ongoing ADT at baseline and the number of ADT failures during the current episode. The post hoc analyses demonstrated that cariprazine (1.5 mg/d and 3 mg/d) + ADT reduced MADRS score significantly compared to ADT+ placebo and was effective in lowering MADRS total score regardless of the level of response to ongoing ADT at baseline or the number of prior ADT failures in the current episode [46].

In a recent post hoc analysis, Thase et al. collected and analyzed the results of all previous clinical trials to evaluate the long-term safety, tolerability, and efficacy of cariprazine as an adjunctive treatment for patients with MDD who had an inadequate response to ADT [47]. In total, results from 2,222 participants who had previously received treatment with cariprazine in a placebo-controlled or open-label study were collected. The results showed that cariprazine is generally safe and well-tolerated. Treatment-emergent AEs occurred in 61% of cariprazine-treated patients and 48% of placebo-treated patients. The most common AEs were akathisia (11% in cariprazine-treated patients and 2% in placebo-treated patients) and restlessness (6% in cariprazine-treated patients and 2% in placebo-treated patients). Discontinuation due to an AE occurred in 6% of cariprazine-treated patients and 2% of placebo-treated patients [47].

The changes in metabolic parameters, including shifts in fasting glucose and lipid parameters, were similar in cariprazine- and placebo-treated patients [47]. In the long-term safety study, the mean weight change was 1.6 kg over six months. Other safety endpoints, including laboratory and C-SSRS assessments of suicidality, were generally consistent with the safety profile of cariprazine in approved indications of bipolar disorder and schizophrenia [47].

While this recent post hoc analysis revealed no new safety signals and the data is consistent with the currently approved prescribing information, as with all medications, the benefits and risks should be carefully evaluated individually. Patients should be closely monitored for AEs during treatment.

Limitations and drawbacks of cariprazine

It is essential to consider the limitations and drawbacks of cariprazine when deciding its use in treating psychiatric conditions. While numerous studies have shown cariprazine to be effective in treating bipolar mania and depression, others have suggested that it may not be a good choice for certain patients or may be associated with adverse effects. For example, a study published in the Journal of Psychopharmacology found that cariprazine may not be effective in treating psychosis associated with Parkinson's disease and may even worsen some symptoms [48]. Additionally, cariprazine may be associated with a risk of akathisia, a condition characterized by restlessness, agitation, and an inability to sit still [40-45,48]. Patients taking cariprazine should be monitored closely for signs of akathisia and other adverse effects. It is crucial to weigh the potential benefits and risks of cariprazine when deciding its use in treating psychiatric conditions. While some patients may benefit from this medication, others may not respond well or experience adverse effects.

While several clinical trials have investigated the safety and efficacy of cariprazine for treating psychiatric disorders, most of these studies have only compared it to a placebo. Consequently, there is a critical need for additional head-to-head trials that directly compare cariprazine to other commonly used SGAs, such as Seroquel or Olanzapine. These studies are essential in establishing the relative effectiveness and safety of cariprazine compared to other frequently prescribed antipsychotic medications. Furthermore, they can provide valuable information to clinicians and patients to make informed decisions about the most appropriate treatment options for their needs.

Head-to-head trials can contribute significantly to developing treatment guidelines and formulary decisions made by healthcare organizations. Although cariprazine has demonstrated promising results in clinical trials against placebo, further research comparing its effectiveness and safety to other SGAs in head-to-head trials is crucial to establish its relative benefits and risks. Therefore, conducting such studies is imperative to improve the current understanding of cariprazine's potential in treating psychiatric disorders and to guide clinical decision-making.

## Conclusions

Mood disorders, including major depression and bipolar disorder, and psychotic disorders, such as schizophrenia, are complex and challenging mental illnesses that can significantly impact an individual's daily functioning and quality of life. While the prevalence of these disorders varies, they are all considered a significant burden to global health. The emergence of atypical antipsychotic agents in the late 1990s revolutionized the management of these disorders, providing a broad-spectrum efficacy previously unavailable. However, these agents have significant concerns regarding their side effect profiles, leading to a shift in focus toward identifying atypical agents with a more favorable profile. Cariprazine is one such agent that has demonstrated a unique mechanism of action and favorable safety profile, making it a promising medication for managing a spectrum of mental health conditions. This review has provided a thorough analysis of cariprazine's efficacy and safety profile, which will be helpful for clinicians and researchers.

## Figures and Tables

**Figure 1:**
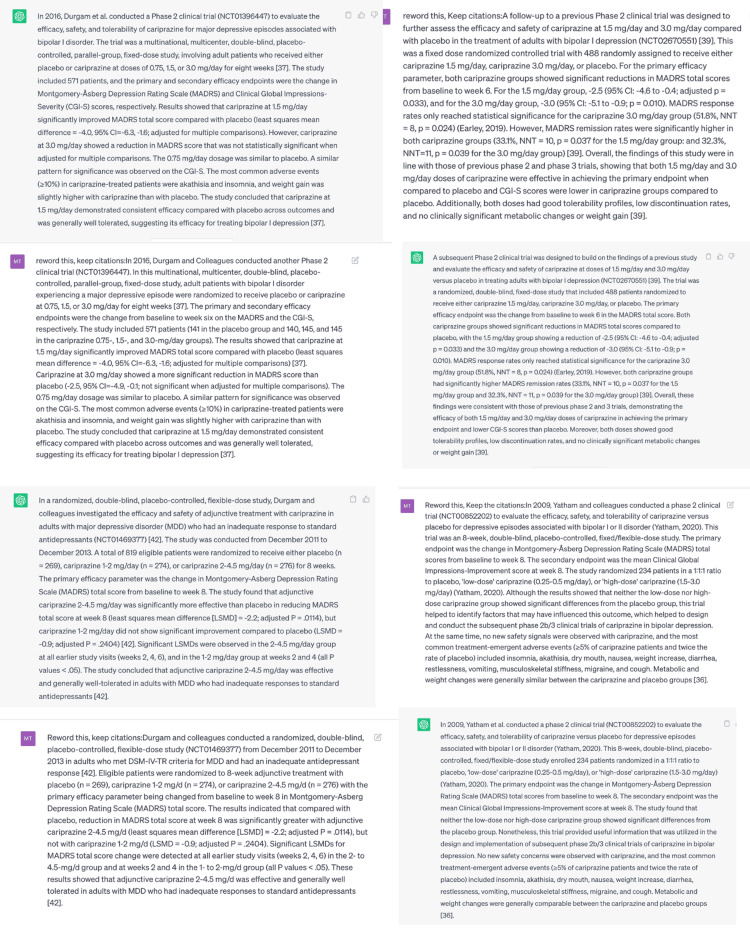
ChatGPT-assisted editing and rewording of the review article

## Appendices

The ChatGPT language model (OpenAI, San Francisco, California) was utilized to assist in editing and rewording the work to ensure coherence and clarity in conveying the findings (Figure 1).
